# Means of Removing the Rancidity of Butter

**Published:** 1854-03

**Authors:** 


*Means of removing the Rancidity of Butter.*—Wild recommends that the butter should be kneaded with fresh milk and then with pure water. He states that by his treatment the butter is rendered as fresh and pure in flavor as when recently made. He ascribes this result to the fact that butyric acid, to which the rancid odor and taste are owing, is readily soluble in fresh milk, and is thus removed.—*Pharm. Journal.*

